# The Kinematics of Swimming and Relocation Jumps in Copepod Nauplii

**DOI:** 10.1371/journal.pone.0047486

**Published:** 2012-10-24

**Authors:** Christian Marc Andersen Borg, Eleonora Bruno, Thomas Kiørboe

**Affiliations:** Centre for Ocean Life, National Institute for Aquatic Resources, Technical University of Denmark, Charlottenlund, Denmark; Institute of Marine Research, Norway

## Abstract

Copepod nauplii move in a world dominated by viscosity. Their swimming-by-jumping propulsion mode, with alternating power and recovery strokes of three pairs of cephalic appendages, is fundamentally different from the way other microplankters move. Protozoans move using cilia or flagella, and copepodites are equipped with highly specialized swimming legs. In some species the nauplius may also propel itself more slowly through the water by beating and rotating the appendages in a different, more complex pattern. We use high-speed video to describe jumping and swimming in nauplii of three species of pelagic copepods: *Temora longicornis*, *Oithona davisae* and *Acartia tonsa*. The kinematics of jumping is similar between the three species. Jumps result in a very erratic translation with no phase of passive coasting and the nauplii move backwards during recovery strokes. This is due to poorly synchronized recovery strokes and a low beat frequency relative to the coasting time scale. For the same reason, the propulsion efficiency of the nauplii is low. Given the universality of the nauplius body plan, it is surprising that they seem to be inefficient when jumping, which is different from the very efficient larger copepodites. A slow-swimming mode is only displayed by *T. longicornis*. In this mode, beating of the appendages results in the creation of a strong feeding current that is about 10 times faster than the average translation speed of the nauplius. The nauplius is thus essentially hovering when feeding, which results in a higher feeding efficiency than that of a nauplius cruising through the water.

## Introduction

Copepod nauplii are ubiquitous, abundant and productive metazoans in the ocean [1]–[2]. Together with protists they dominate the microzooplankton, which are the main consumers of the oceans’ primary production [3]. Here we report on the motile behavior of copepod nauplii.

Both protists and nauplii swim in a low Reynolds number, viscous world. Protists typically swim by beating flagella or by metachronal waves of cilia, and the kinematics and hydrodynamics of protist swimming are rather well described and understood [4]–[9]. The swimming in protists is typically smooth because of the high beat frequencies [10] and/or because they possess flagella with helical beat patterns that, similar to a propeller, yields nearly time-invariant propulsion force [5]–[7], [11]. Most protists swim in a helical pattern, both because of the propulsion asymmetry imposed by the arrangement of the flagella/cilia [5]–[6], but also because any asymmetry at low Reynolds numbers will cause rotation that may lead to a helical swimming path [12]–[13]. Copepod nauplii are bilaterally symmetrical crustaceans with an exoskeleton onto which the muscular apparatus attaches [14] and they swim or jump by means of appendages in a way fundamentally different from protists. Both nauplii and copepodites may display three basic modes of motile behavior: 1) motionless sinking, 2) swimming by vibrating and rotating the feeding appendages at a high frequency, or 3) swimming-by-jumping, which is conducted by alternating power and recovery strokes of the appendages [15]–[21]. Many nauplii and copepods can only move in the latter mode. Propulsion during swimming is relatively slow and commonly described as smooth, while swimming-by-jumping results in much higher instantaneous velocities and unsteady motion, even in copepods where the influence of inertia may be significant [21]–[24]. High speed observations and fluid mechanical models of swimming-by-jumping in copepods show that this motility mode is energetically very efficient [24]–[25]. Similar information is not available for nauplii.

Nauplii are smaller than copepodites and adult copepods, and consequently operate at much lower Reynolds numbers. Moreover, while copepodites have six pairs of cephalic appendages used for swimming and food collection and up to five pairs of specialized “swimming legs” allocated for jumping, nauplii have only three pairs of appendages to be used for motion and feeding. We therefore expect the kinematics of nauplii to be different from that of adults, and it should also be different from that of protists that use flagella or cilia.

Except for Williams [26], who did not provide detailed quantitative information, previous video recordings of nauplii motility have been conducted at frame rates of only 15 to 250 Hz. This is adequate for revealing overall patterns of swimming [27]–[31] and appendage movements [20] (swimming of large *Eucalanus* nauplii), overall time budgets [28]–[33] and for relatively rough determinations of behavioral parameters such as average velocities [27]–[31], [33]–[35] and prey handling time [31]. However, the details of appendage movements and velocity patterns require a higher recording speed.

Here, we describe the kinematics of relocation jumps in nauplii of three species of marine copepods, *Temora longicornis*, *Oithona davisae* and *Acartia tonsa*, and the kinematics of slow swimming in *T. longicornis*, by the use of high-speed video technique. The motion of adults of these species has been fairly well described as has the overall swimming behavior and time budget of the nauplii. The three species represent three different overall behavioral patterns. In *T. longicornis*, nauplii, slow swimming alternates with motionless sinking and occasional relocation jumps [29], [32], while in *A. tonsa* there is no such slow swimming but instead frequent relocation jumps [29], [36]. In *O. davisae*, long periods of motionless sinking are interrupted only by infrequent jumps [31]. To our knowledge, the present study is the first attempt to describe in detail the kinematics of jumping and swimming in copepod nauplii. We include all naupliar stages of the 3 species, ranging in size from 0.1 to 0.4 mm, and compare the size scaling of kinematics between them.

## Methods

Nauplii were collected from our continuous cultures kept at 30 PSU and 14°C (*T. longicornis* and *A. tonsa*) or 22°C (*O. davisae*), gently rinsed with 0.2 µm filtered sea water (30 PSU) and transferred to either 60 ml polycarbonate bottles (*T. longicornis* and *A. tonsa*) or 4 ml glass cuvettes (*O. davisae*) for observations. Observational containers contained a suspension of either *Rhodomonas salina* (cryptophyte, equivalent spherical diameter, ESD ∼ 7.6 µm, used as prey for *T. longicornis* and *A. tonsa*), *Heterocapsa triquetra* (dinoflagellate, ESD ∼ 14.3 µm, used for *T. longicornis*) or *Oxyrrhis marina* (dinoflagellate, ESD ∼ 16.9 µm, used for *O. davisae*) and the nauplii were allowed to acclimate for 1–2 hrs before filming in a temperature-controlled room at 16°C (*T. longicornis* & *A. tonsa*) or 22°C (*O. davisae*).

Jumps and swimming bouts (for *T. longicornis*) were recorded with a high-speed digital video camera, Phantom v. 210, at a resolution of 1024 × 768 pixels (*T. longicornis*) or 1024 x 800 (*A. tonsa* & *O. davisae*), frame rates of 2000 or 2200 frames s^−1^, and fields of view of 7.8 or 31.4 mm^2^. Illumination was provided by a halogen bulb pointed into the experimental container towards the camera. For observations in 60 ml bottles the light was passed through a collimator lens. Exposure times ranged from 150 to 490 µs. Only recordings where the nauplius was moving in the focal plane perpendicular to the direction of observation were analyzed. The camera software was used to make accurate measurements of nauplii body length (excluding caudal armature), body width (max) and length of the antennules (measured in their resting position and from the dorsal side). Images were calibrated by filming spheres of known diameter.

We used the freeware ImageJ to digitize the temporal positions of the body and of the appendages. Temporal positions of the mean of the front tip and end of body (discounting the caudal armature) were used to calculate velocities. In some cases we also digitized the tips of either the left or the right antennule, antenna and mandible – hereafter named A1, A2 and Md, respectively. These were positioned in a coordinate system with the tip of the head as the origin and the x-axis aligned with the length of the body. A total of 63, 82 and 82 spontaneous relocation jumps were fully analyzed for *T. longicornis*, *O. davisae* and *A. tonsa*, respectively. In addition, we analyzed 71 swimming sequences of *T. longicornis*. For all three species the nauplii analyzed covered the full size range from nauplii stage 1 to 6 (N1 to N6) (Table 1). All distances are measured in two-dimensional projections and therefore conservative.

**Table 1:** Characteristics of the experimental nauplii and their repositioning jumps and swimming.

		No. BC’s per jump		Total jump dist.	Initial turn characteristics			Power stroke delay, ms		Recovery stroke delay, ms		Beat phase duration	
Species	BL, µm	average	Range	BL’s	occur., %	durat., ms	angle, deg.	Md→A2	A2→A1	Md→A2	A2→A1	ms	% of total BC
*T.l.*, jump	97–382	2.8±1.9	1–9	*range: 1–16	56	6.7±0.7	**range: 11–58	1.1±0.4	3.0±0.5	0.3±0.5	0.7±0.4	9.1±1.1	80.5±4.0
*T.l*, swim	149–357	n.a.	n.a.	n.a.	n.a.	n.a.	n.a.	ca. 12	ca.12	ca.12	ca.12	14.2±2.4	57.2±8.1
*O.d*., jump	86–174	3.8±1.3	2–8	4.2±0.2	26	5.1±0.9	49.2±8.9	1.3±0.4	1.0±0.2	0.4±0.2	0.9±0.2	5.0±0.6	77.4±3.0
*A.t.*, jump	117–337	2.0±1.1	1–7	2.0±1.1	0	n.a.	n.a.	0.5±0.3	2.0±0.3	0.0±0.1	1.8±0.6	11.4±2.6	72.2±4.6

*T.l.*: *Temora longicornis*, *O.d.*: *Oithona davisae*, *A.t.*: *Acartia tonsa*, BL: body length, BC: beat cycle,, A1: antennules, A2: antennae, Md: mandibles, n.a.: not applicable. Values are given ± SD. *Log(TJD) = −0.98Log(BL_mm_) –0.17 (n = 53, r^2^ = 0.35, p<000.1),

** Log(ANG)  = −0.57Log(BL_mm_) +1.12 (n = 40, r^2^ = 0.56, p<0.0001), TJD: total jump distance, ANG: angle of initial turn.

## Results

### Nauplii Size and Allometry

**Figure 1:**
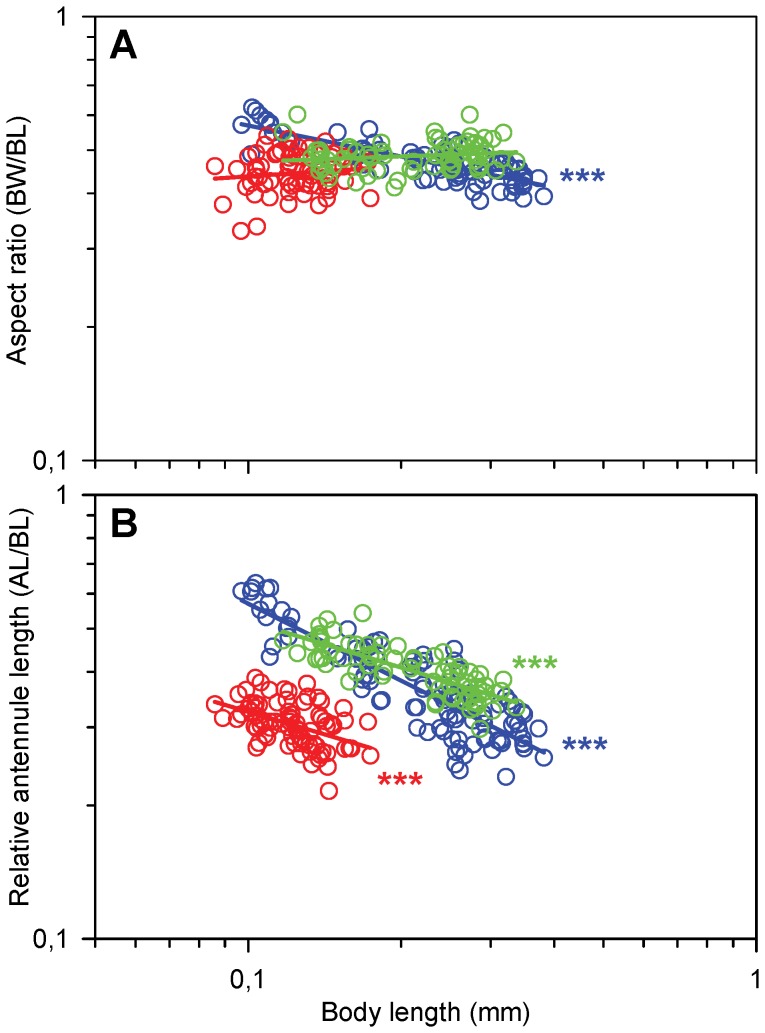
Morphology as function of body length (BL) in nauplii. Regression lines are power laws fitted to the data. A) Aspect ratio (AR, body width/body length). Blue circles, *T. longicornis,* power law equation: Log(AR)  = −0.23Log(BL) –0.48 (r^2^ = 0.69, p<0.0001, n = 88); red circles, *O. davisae* (slope  = 0.07, p = 0.47, n = 64); green circles *A. tonsa*, (slope  = 0.04, p = 0.10, n = 70), B) Relative antennule length (RAL, antennule length/body length). Blue circles, *T. longicornis*: Log(RAL)  = −0.58Log(BL) –0.82 (r^2^ = 0.75, p<0.0001, n = 100); red circles, *O. davisae*: Log(RAL)  = −0.34Log(BL)–0.83 (r^2^ = 0.21, p<0.0001, n = 83); green circles, *A. tonsa*: Log(RAL)  = −0.35Log(BL)–0.63 (r^2^ = 0.63, p<0.0001, n = 71).

We found marked differences in the development of body shape and relative length of appendages between the three species (Fig. 1). The nauplii of *T. longicornis* become progressively slimmer as they grow and the body aspect ratio (body width to body length) decreases from ca. 0.6 to 0.4. In contrast, the nauplii of the two other species have size-independent aspect ratios of 0.49 (*A. tonsa*) and 0.45 (*O. davisae*).

The nauplii have three pairs of appendages that are all involved in propulsion: The antennules (A1), the antennae (A2), and the mandibles (Md). In the later naupliar stages a fourth pair of appendages (maxillulae) as well as the rudiments of the fifth pair (maxillae) appears, but neither of them are functionally important. The relative antennule length (antennule length/body length) decreases strongly with body length in all three species; most pronounced in *T. longicornis* (Fig. 1B). At any given body length the relative antennule length in *O. davisae* is only about 50% of that in the two other species.

### Description of Jumps and Swimming

**Figure 2:**
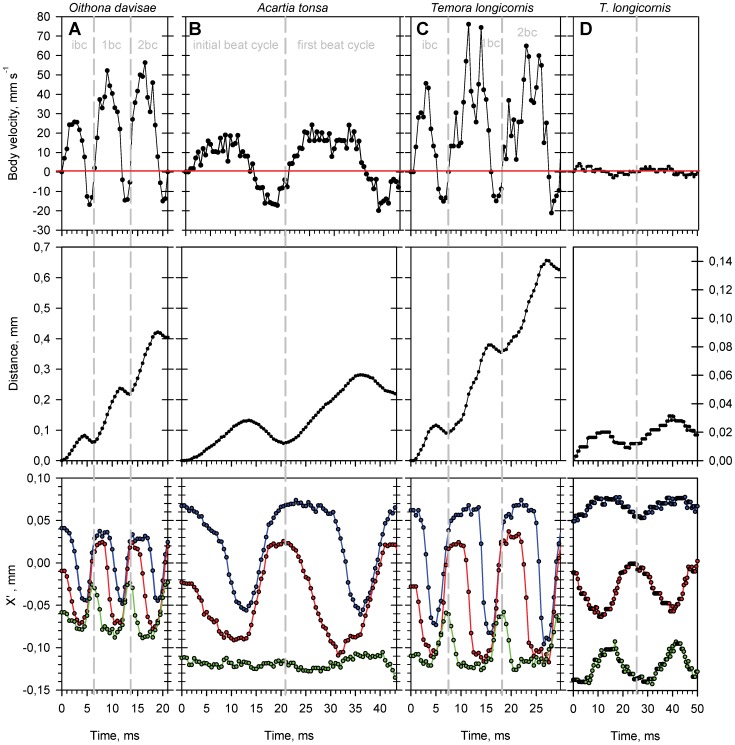
Characteristics of spontaneous relocation jumps and of swimming. Beat cycles analyzed for an individual nauplius of: A) *Oithona davisae* (body length = 0.154 mm), B) *Acartia tonsa* (body length = 0.154 mm), C) *Temora longicornis* (body length = 0.168 mm), and D) *T. longicornis* swimming (body length = 0.290 mm). Body velocity (upper panels), total distance travelled (middle panels) and the position of appendages relative to the tip of the nauplius head (lower panels), all as function of time. Positions of the tips of antennules, antennae and mandibles relative to the tip of the body are shown in blue, red and green, respectively. Grey vertical dashed lines indicate the end/beginning of a beat cycle, ibc: initial beat cycle, 1bc: first beat cycle, 2bc: second beat cycle.

The three species are roughly similar in the way they jump (Fig. 2, Video S1, S2, S3). In *Temora longicornis* the jump in N2–N6 may be initiated from either the swimming mode (52%) or the motionless sinking mode (48%). The N1 of *T. longicornis* as well as all stages of *Oithona davisae* and *Acartia tonsa* do not display swimming behavior, and hence the jump is always initiated from motionless sinking. In all three species the jumping track may look anything from almost perfect straight to somewhat curved, and the animal may rotate around its longitudinal axis.

The jump starts by the backward strokes of A1 followed by A2; only in *O. davisae* Md also sometimes strike backward. The subsequent recovery stroke may be synchronized or include a short phase delay between the appendages. The duration of the whole “initial beat cycle” is 7–8 ms in *T. longicornis* (increasing with size), 4–7 ms in *O. davisae* (independent of size) and 11–21 ms in *A. tonsa* (decreasing with size). After the initial beat cycle follows up to several intermediate beat cycles (Table 1, Fig. 2). In *T. longicornis* and *O. davisae* all three functional appendages are involved, while in *A. tonsa* only A1 and A2 are important for propulsion; the mandibles move only little or not at all. In all three species the appendages move constantly in a metachronal wave during both the power and the recovery strokes and for that reason there is no distinct separation between the beat phase and the recovery phase and no gliding phase like that described for copepodites [23]. Nevertheless, there is a distinct pattern of alternating positive and negative body velocities associated with each beat cycle giving the whole jump its characteristic “jerky” appearance (Fig. 2). By our definition the beat phase occurs when body velocity is positive and the recovery phase occurs when body velocity is zero or negative. Propulsion is gained from the asymmetry between power and recovery strokes: During the power stroke the appendages and setae are more or less straight and spread out like a fan to maximize the surface area, while during the recovery the appendages are slightly bent and the setae strongly bent backwards with the “fan” more or less collapsed (Video S1, S2, S3). Moreover, the phase delay between appendages is shorter during the recovery than during the beat phase (Fig. 2, Table 1).

After the last beat cycle the appendages are brought backwards from their anterior-most position to the resting position, while the nauplius moves slowly forward and eventually stops. The duration of this finishing phase is about 18 ms for *T. longicornis*, 11 ms for *A. tonsa* and 9 ms for *O. davisae* (independent of body length, data not shown). Alternatively, in *T. longicornis* (but not in *O. davisae* and *A. tonsa*), swimming (i.e. feeding current movements) may commence immediately after the last beat cycle.

Nauplii of *T. longicornis* and *O. davisae*, but not *A. tonsa*, may perform an initial turn around the dorsoventral axis and thereby reorient before jumping forward (see Table 1). The turn is accomplished by a backward stroke with the appendages on one side together with a simultaneous countermovement of the opposite appendages, followed by a recovery stroke that brings all appendages to their anterior-most resting position. In *A. tonsa* reorientation takes place during the jump and is accomplished by slightly asymmetric strokes. Angular speed during the turn is higher in *O. davisae* (ca. 10 deg. ms^−1^) than in *T. longicornis* of the same size (6–7 deg. ms^−1^, Table 1).

All nauplii stages of *T. longicornis*, except the N1, may also “swim”, i.e., move forward while creating a feeding current by rotating and/or beating all three pairs of appendages in a continuous metachronal rhythm (Video S4). As evident in the jump, the swimming beat cycle contains a beat phase when body velocity is positive and a recovery phase when body velocity is negative (Fig. 2, Table 1). However, the beat amplitudes of especially A1 and A2 are much smaller than in the case of jumping and the beat patterns are more complex, containing rotational elements not displayed during jumping. Forward propulsion is gained from the power stroke of A2, while the A1 and Md are in counter-phase with the A2. Moreover, the A2 does not bend and collapse during the recovery stroke to minimize drag, as observed during jumping. The resulting swimming velocity is much lower than jump velocity (Fig. 2).

### Beat Cycle Frequency

**Figure 3:**
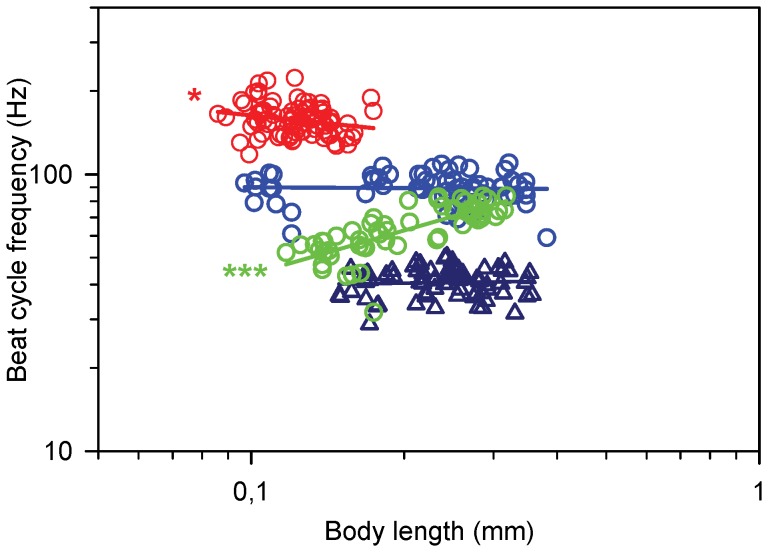
Beat cycle frequency (BCF) as function of body length (BL) in nauplii. Regression lines are power laws fitted to the data. Blue circles, *T. longicornis* (slope  = −0.01, p = 0.53, n = 63); red circles, *O. davisae*: Log(BCF) = −0.19Log(BL) +2.02 (r^2^ = 0.05, p = 0.046, n = 82); green circles, *A. tonsa*: Log(BCF)  = 0.53Log(BL) +2.17 (r^2^ = 0.57, p<0.0001, n = 59); blue triangles, swimming *T. longicornis* (slope  = 0.03, p = 0.89, n = 71).

There are substantial differences in beat cycle frequency, beat phase duration, and scaling with body size between the three species. In *T. longicornis* the beat cycle frequency is independent of body length and approximately 90 Hz (Fig. 3). *O. davisae* has a beat cycle frequency almost twice as high and it decreases slightly with body length. In contrast, the beat cycle frequency of *A. tonsa* increases strongly with body length, from ca. 50 to ca. 80 Hz. Differences between species are not closely related to body size. The relative duration of beat and recovery phases are independent of body size but vary between species (Table 1). Beat cycle frequency for swimming *T. longicornis* is independent of body length and on average ca. 40 Hz or less than half the frequency during jumping (Fig. 3).

There is some variation between species in the phase delay between the appendages in the power stroke, and phase delays are independent of body length. In *T. longicornis* there is ca. 1 ms between the beating mandibles and antennae and 3 ms between the antennae and antennules (Table 1, Fig. 2A); in *A. tonsa* ca. 0.5 and 2 ms, respectively (Fig. 2C). In *O. davisae* the beat sequence is more symmetrical and the phase delays are both ca 1.0 ms (Fig. 2B). The phase delay during appendage recovery is longest in *A. tonsa* (0.0 and 1.8 ms) and shorter in *T. longicornis* (0.3 and 0.7 ms) and *O. davisae* (0.4 and 0.9 ms). Appendage phase delays for swimming *T. longicornis* are symmetrical and ca. 12 ms (Table 1, Fig. 2D).

### Distance Per Beat Cycle & Total Jump Distance

**Figure 4:**
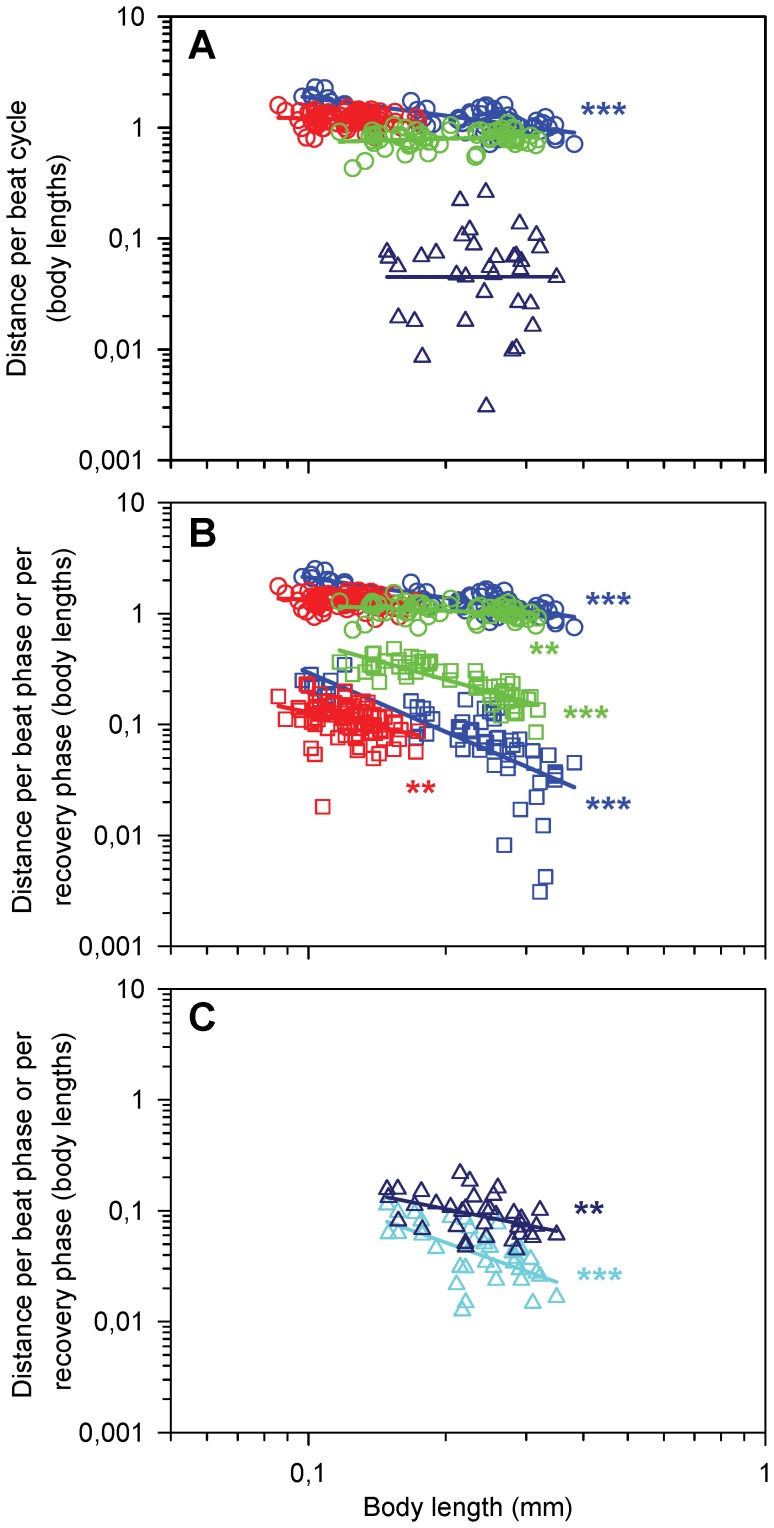
Distance travelled per beat as function of body length (BL) in nauplii. Regression lines are power laws fitted to the data. A) Body length specific net distance per beat cycle (DBC). Blue circles, *T. longicornis*: Log(DBC) = −0.54Log(BL) –0.27 (r^2^ = 0.58, p<0.0001, n = 63); red circles, *O. davisae* (slope  = −0.07, p = 0.29, n = 82); green circles, *A. tonsa* (slope  = 0.12, p = 0.25, n = 59); blue triangles, swimming *T. longicornis* (slope  = 0.01, p = 0.94, n = 36), B) Body length specific distances for the beat (DBP) and recovery (DRP) phases. Blue circles, *T. longicornis* beat phase (slope  = −0.61, r^2^ = 0.70, p<0.0001, n = 63); blue squares, *T. longicornis* recovery phase (slope  = −1.77, r^2^ = 0.54, p<0.0001, n = 63); red circles, *O. davisae* beat phase (slope  = −0.16, r^2^ = 0.03, p = 0.08, n = 81); red squares, *O. davisae* recovery phase (slope  = −0.86, r^2^ = 0.11, p = 0.001, n = 81); green circles, *A. tonsa* beat phase (slope  = −0.17, r^2^ = 0.09, p = 0.006, n = 59); green squares, *A. tonsa* recovery phase (slope  = −1.14, r^2^ = 0.70, p<0.0001, n = 58), C) Swimming *T. longicornis*, body length specific distances for the beat (DBP) and recovery phases (DRP). Blue triangles, beat phase: Log(DBP)  = −0.82Log(BL) –1.56 (r^2^ = 0.22, n = 36, p = 0.005); cyan triangles, recovery phase: Log(DRP)  = −1.47Log(BL) –2.32 (r^2^ = 0.36, n = 36, p<0.0001).

The beating of the appendages propels the animal forward, but the relative net distance covered per beat cycle varies, between 0.8 to 2.0 body lengths for jumps (Fig. 4A). We found the longest specific distances in *T. longicornis* and a decrease in relative distance with increase in size, from ca 2 to 1 body lengths. In *O. davisae* and *A. tonsa* the specific distances were shorter, 1.2 and 0.8 body lengths per beat cycle, respectively, and independent of size. For swimming *T. longicornis* the net distance covered per beat cycle was highly variable, but independent of size, and averaged only 0.06 body lengths (Fig. 4A).

More detailed information may be gained if the full beat cycle is broken down into its power and recovery phases (Fig. 4B, C). When jumping, the distance covered per power stroke are almost similar between species, while there are pronounced differences in the backwards distances moved during the recovery phase. The specific backward recovery distances decline with body size in all three species, but are much longer in *A. tonsa* than in the other species, 0.15–0.50 body lengths per recovery (10–40% of the corresponding distance per power stroke), which is two to three times longer than in *T. longicornis* (2–12%) and ca four times longer than in *O. davisae* (5–10%) (Fig. 2, 4B). Thus, the net forward propulsion appears to be inefficient in *A. tonsa* relative to the other species. In swimming *T. longicornis* the specific backward recovery distances were much higher than for jumping: ca 50% of the corresponding distance per power stroke (Fig. 2, 4C).

The total number of beat cycles per jump, including the initial cycle, ranged from 1 to 9 and was not related to size in any of the three species (Table 1). The relative jump distance decreased with size in *T. longicornis* from ca. 7 to 2 body lengths per jump (Table 1). In *A. tonsa* and *O. davisae* the relative distance was independent of size and on average around 1.6 and 4.2 body lengths per jump, respectively (Table 1), but with great variation in both species. We were not able to obtain data for complete swimming bouts in *T. longicornis*.

### Maximum and Average Velocities

**Figure 5:**
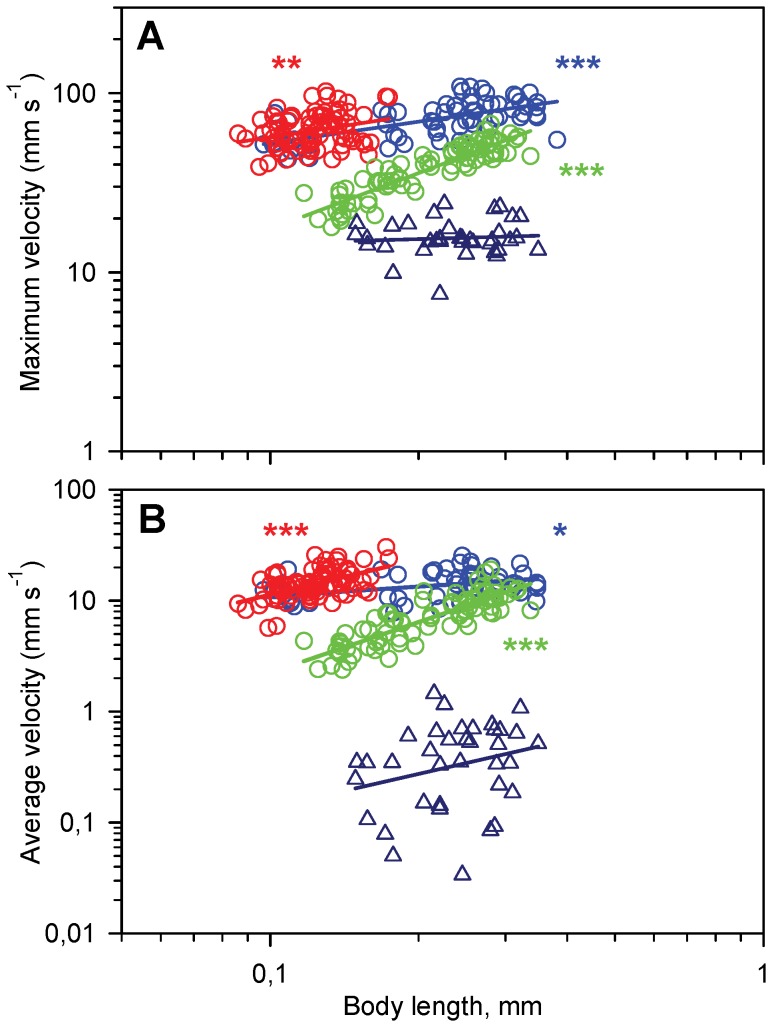
Jumping and swimming velocities of nauplii as a function of body length (BL). Regression lines are power laws fitted to the data. A) Maximum velocity (max vel). Blue circles, *T. longicornis*: Log(max vel)  = 0.40Log(BL) +2.119 (r^2^ = 0.40, p<0.0001, n = 63); red circles, *O. davisae*: Log(max vel)  = 0.42Log(BL) +2.178 (r^2^ = 0.08, p = 0.005, n = 82); green circles, *A. tonsa*: Log(max vel)  = 1.04Log(BL) +2.279 (r^2^ = 0.82, p<0.0001, n = 82); blue triangles, *T. longicornis* swimming (slope  = 0.08, p = 0.58, n = 36), B) Average velocity (avg vel), calculated for the entire relocation jump. Blue circles, *T. longicornis*: Log(max vel)  = 0.30Log(BL) +1.334 (r^2^ = 0.14, p = 0.01, n = 54); red circles, *O. davisae*: Log(max vel)  = 1.06Log(BL) +2.116 (r^2^ = 0.29, p<0.0001, n = 82); green circles, *A. tonsa*: Log(max vel) = 1.52Log(BL) +1.869 (r^2^ = 0.73, p<0.0001, n = 82); blue triangles, *T. longicornis* swimming (slope  = 1.01, p = 0.18, n = 36).

Maximum jump velocity (mm s^−1^) increases with body length in all three species (Fig. 5A), while maximum relative velocity (body lengths s^−1^) decreases; for *T. longicornis* from ca. 550 to 250 body lengths s^−1^. Similar values (a decrease from ca. 600 to 400 body lengths s^−1^) were recorded for *O. davisae*, which is significantly higher than for *A. tonsa* where maximum relative velocitwas ca. 190 body lengths s^−1^. In swimming *T. longicornis* maximum velocity is independent of body length and ca. 16 mm s^−1^ (ca. 40–100 body lengths s^−1^).

Average velocities (mm s^−1^) calculated for the entire jump increase with body length in all three species (Fig. 5B). The average relative velocity (body lengths s^−1^) decreases with body length from ca. 170 to 80 body lengths s^−1^ in *T. longicornis* and from ca. 200 to 160 body lengths s^−1^ in *O. davisae* but increases in *A. tonsa*, from ca. 30 to 60 body lengths s^−1^ (Fig. 5B). The rather large variability around the regression lines are partly due to a large individual variability in the number of beat cycles per jump (Table 1), and thus in the relative importance of the slower final phase of the jump. For the same reason the values are 12–64% lower than corresponding average velocities calculated for the intermediate beat cycles only (data not shown). Average swimming velocity in *T. longicornis* was 0.44 mm s^−1^ but varied greatly (±0.32, range: 0.03–1.23; Fig. 5) and was dependent more on the directionality of the swimming (the effect of gravity) than on naupliar size. We observed that nauplii swimming “upwards” would almost be hovering.

**Figure 6:**
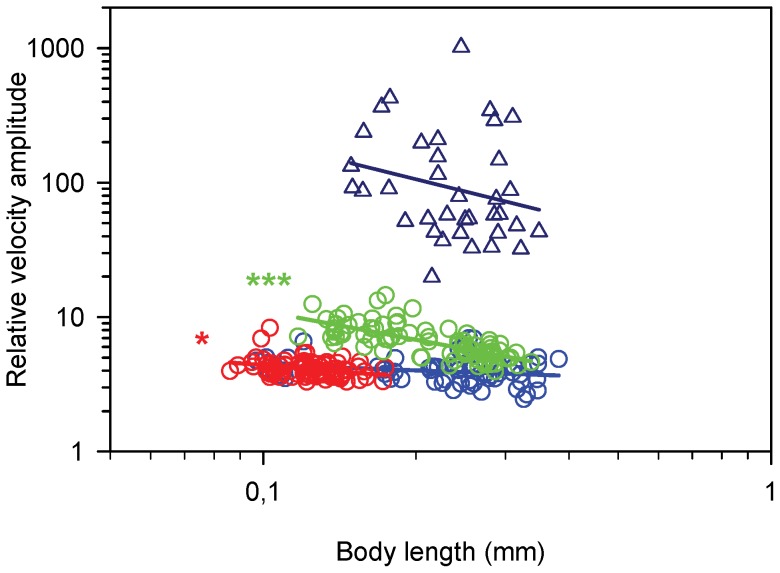
Relative velocity fluctuation (RLF), as function of body length (BL) in nauplii. RLF  =  (maximum velocity – minimum velocity)/average velocity). Regression lines are power laws fitted to the data. Blue circles, *T. longicornis*: Log(RLF)  = −0.13Log(BL) +0.51 (r^2^ = 0.06, p = 0.12, n = 63); red circles, *O. davisae*, Log(RLF)  = −0.31Log(BL) +0.34 (r^2^ = 0.10, p = 0.01, n = 82); green circles, *A. tonsa*, Log(RLF)  = −0.71Log(BL) +0.34 (r^2^ = 0.50, p<0.0001, n = 82); blue triangles, *T. longicornis* swimming (slope  = −0.93, p = 0.49, n = 36).

The relative velocity fluctuation during a beat cycle – which is a way of expressing the degree of jump “smoothness” - is substantial and varies significantly with body size and between species (Figs 2 and 6). In jumping nauplii the fluctuation declines with size in all species but it is much larger in *A. tonsa* than in the other species. Interestingly, the relative velocity fluctuation in swimming *T. longicornis* is an order of magnitude higher than for jumping.

## Discussion

### Morphology and Allometry

The free-swimming nauplius is phylotypic and a fundamental developmental constraint for all crustaceans [26], so despite the taxonomic difference between the two calanoids (*T. longicornis* and *A. tonsa*) and the cyclopoid (*O. davisae*) [37]–[39], the nauplii of the three species share the same body plan. The relative similarity in body shape between nauplii – a slightly elongated sphere, different from the more elongated body form of the copepodites – suggests that it is optimized for motility or other purposes.

The Reynolds number calculated for average jump velocities ranges from 0.3 for the smallest and slowest to 6 for the largest and fastest nauplii examined here. The optimal body shape for swimming at Reynolds numbers <1 in terms of minimizing the drag resistance is that of an ellipsoid with an aspect ratio of 0.53 [40]; this optimum decreases with size to about 0.4 at Re 6 (Uffe H. Thygesen [Unpublished]). For escape jumps the velocity and thus Re would be higher (for *T. longicornis* and *A. tonsa* ca. four and eight times higher, respectively, [33] and the optimal aspect ratio lower (ca 0.3, Uffe H. Thygesen, [Unpublished]). Thus, the aspect ratio of the body of the smallest nauplii, and the decrease in aspect ratio with increasing size found in *T. longicornis* may be adaptations to resistance minimization during jumping. The advantage of optimal shape to the nauplius is maybe not so much to minimize energetic costs that are assumed to be low [41]–[42], but to allow the highest possible speed during rapid escape and prey attack jumps (*A. tonsa* and *O. davisae*, Bruno E, Andersen Borg CM, Kiørboe T. Prey detection and prey capture in copepod nauplii [Unpublished]). The more slender body of copepodites (aspect ratio below 0.4 if one includes the telson) is consistent with this interpretation. In comparison, the body shape of barnacle nauplii (Cirripedia), which are slow swimmers exhibiting no or only weak escape responses [43]–[44], is very far from streamlined. Their aspect ratios, even excluding the stout fronto-lateral horns, lie generally in the range of 0.6 to 0.7 [45]–[46].

### Kinematics of Jumping

The free-swimming nauplius is functionally plastic and modes of locomotion may differ significantly, even between closely related crustacean species [26]. However, the three described copepod species are roughly similar in the way they jump. The kinematics of nauplii jumping differ from those of the copepodites and adults (*T. longicornis* [21], [47], *A. tonsa* [22]–[23], [48]–[49], *O. davisae*: [23]–[24], [48]). First of all, copepodites are equipped with five pairs of cephalic appendages allocated for swimming, feeding and sensory functions, and four or five pairs of thoracic appendages (“swimming legs”) specialized for jumping, while the nauplii have only three functional pairs of appendages. In copepodites the thoracic appendages move metachronally only during the power stroke, while the recovery stroke is synchronized. In the nauplii the recovery strokes are never perfectly synchronized and there is always a phase delay between the appendages, particularly in *A. tonsa* (Fig. 2C, Table 1).

Because of the poorly synchronized recovery stroke and due to the low Reynolds number of the jumps, the swimming pattern of the nauplii becomes very erratic, with the nauplius even moving backwards during the recovery stroke (Figs. 2 & 4B). Forward propulsion at low Re numbers is only possible because of the asymmetry between the beat and recovery phases. During the beat phase the appendages and setae are more or less straight and spread out like a fan to maximize the surface area, while during recovery the appendages, particularly the setae, bend backwards with the “fan” more or less collapsed. The same pattern of reducing drag by spreading and collapsing setae has been described for nauplii of *Calanus finmarchicus* [17] as well as for the swimming legs of adult *C. finmarchicus* [50]. Moreover, in the nauplii the phase delay between appendages is shorter during recovery than during the beat phase (Fig. 2, Table 1).

Also copepodites move erratically when jumping, but even small *Oithona davisae* (Re_avg_ = 15, Re_max_ = 30, [23] of approximately the same size as the largest nauplii in the present study have a distinct coasting phase, where they utilize inertia to glide forward even during the recovery phase, and the coasting duration is well predicted by inertia [23]. This suggests that leg recovery in copepodites produces very little resistance and counter force. The stopping time for a small nauplius can be estimated from the Stokes time scale, *R* ^2^/*ν*, where *R* is the equivalent radius of the nauplius and *ν* the viscosity. The Stokes time scale spans from 1 ms in the smallest to 10 ms in the largest nauplii. However, during the recovery stroke, the nauplius stops much sooner and then moves backwards. Thus, the recovery stroke in the nauplii is not so well adapted for forward swimming as in the copepodites.

The degree of jump ‘smoothness’ is governed by the magnitude of the beat time scale relative to the Stokes time scale. *Temora longicornis* nauplii have a beat time scale around 10 ms, (Fig. 3), and for the largest ones this is similar to the stokes time scale. These nauplii backed very little during the recovery stroke (Fig. 4B), and had the lowest relative velocity amplitude (Fig. 6). In the other end, nauplii of *A. tonsa* had a longer beat time scale and a very erratic swimming pattern with inefficient appendage recovery and pronounced backing (Figs. 4B & 6). In contrast the adults of *O. davisae* and *A. tonsa* have much smoother jumps with relative velocity amplitudes about one order of magnitude lower than the nauplii [23]. Nauplii of other crustaceans, e.g., *Balanus improvisus*, *Artemia salina*, *Eubranchipus vernalis* and *Triops longicaudatus*, have beat frequencies in the order of only 10 Hz, and all appear to have highly erratic and inefficient swimming [17], [26]–[27]. In contrast, many protists, swim more smoothly than copepod nauplii because they have very high beat frequencies compared with the Stokes time scale, and much better adapted recovery strokes [10], [51]. But there are also examples of protists with erratic swimming, for instance, *Chlamydomonas reinhardtii* (body diameter of 7–10 µm and beat frequency of 50–60 Hz, [52]–[53].

Thus, nauplii appear to be very inefficient jumpers, in contrast to the copepodites. One feature that makes jumping very efficient in copepodites, is the unusually high propulsion efficiency that they can achieve, >90% ([25]. This is accomplished by the formation of viscous vortex rings as they jump. Such rings only form if the duration of the power stroke is short relative to the viscous time scale of the fluid disturbance that they generate ([24]–[25]. This is expressed in the “jump number”:
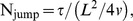
where *τ* is the duration of the power stroke and *L* is the body length. The condition for the formation of viscous vortex rings and, hence, a high propulsion efficiency, is that N < <1. The calculated jump numbers decrease with size, in *T. longicornis* from 3.4 to 0.2, in *O. davisae* from 2.4 to 0.6, and in *A. tonsa* from 2.9 to 0.4. Thus, jumping nauplii do not form viscous vortex rings, and their propulsion efficiency is likely to be a few percent, such as is characteristic for high-jump number swimming at low Re ([51]. This implies that the energetic cost of jumping in nauplii is relatively much higher than in copepodites, especially in the small nauplii.

Given the universality of the nauplius body plan, it may be surprising that they perform so poorly as swimmers. Nauplii of other crustaceans perform even worse, e.g., Cirripedia [43], and *Artemia* nauplii use only the antennae for propulsion; they consequently swim order(s) of magnitude slower. This is in contrast to the copepodite body plan that in many respects appears particularly well adapted to a planktonic life: The muscle-filled torpedo-shaped body and well-coordinated appendage movement allow very high escape speeds, a feature that is considered key to the success of copepods in the ocean [54].

### Kinematics of Swimming in *Temora Longicornis*

In addition to jumping, the nauplii of *T. longicornis* may display a slow-swimming mode. Although carried out by the same three pairs of appendages, this motility mode is fundamentally different from jumping and comprises much more complex beat patterns. Slow swimming results in very erratic translation and forward propulsion is therefore inefficient. The main purpose of the slow swimming mode is to create a feeding current. The nauplii of *A. tonsa* and *O. davisae* are ambush feeders that detect their prey remotely and employ “attack jumps” (Bruno E, Andersen Borg CM, Kiørboe T. Prey detection and prey capture in copepod nauplii [Unpublished]). Translation velocity in *T. longicornis* is only a few tenths of mm s^−1^ and at least an order of magnitude smaller than the feeding current velocity (>3 mm s^−1^, Bruno E, Andersen Borg CM, Kiørboe T. Prey detection and prey capture in copepod nauplii [Unpublished]).

Thus, these nauplii are essentially hovering while feeding. This is likely achieved through the counter phase beating and rotation of the appendages. It is well documented that hovering is more efficient than cruising through the water, both in terms of energy expenditure per volume of water scanned [55] and in terms of volume water cleared per unit force produced [56]. Thus, the nauplii of *T. longicornis* nauplii appear to have optimized their feeding efficiency. There may, however, be a cost to this feeding behavior, because the spatial extension of the fluid disturbance generated by hovering is substantially larger than that generated by cruising through the water and, thus, the vulnerability of the feeding nauplii to rheotactic predators may be elevated [57].

### Concluding Statement

Nauplii have only three pairs of functional appendages, and the species investigated here appear to perform poorly when swimming-by-jumping. This seems also to be the case for a number of other crustacean nauplii, and is in sharp contrast to copepodites that are equipped with specialized swimming legs and are highly efficient swimmers capable of obtaining very high escape speeds. Nauplii of the three species investigated also perform escape jumps, and it remains to be investigated if these are more efficient than relocation jumps. That could be achieved by higher beat frequencies and better coordinated beat patterns. The nauplii of *A. tonsa* and *O. davisae* are ambush feeders – a feeding mode that requires fast and efficient jumping to catch the prey. Nauplii of *T. longicornis*, on the other hand, seem to have optimized their feeding efficiency by creating a strong feeding current while being propelled only slowly through the water. This motility mode is carried out with the same appendages as jumping, but it is fundamentally different.

## Supporting Information

Video S1 **Example jumps of** ***Temora longicornis*** **nauplii played in slow motion X250.** Body lengths: #1: 0.101 mm, #2: 0.346 mm.
(WMV)

Video S2 **Example jumps of** ***Oithona davisae*** **nauplii played in slow motion X250.** Body lengths #1: 0.133 mm, #2: 0.131 mm, #3: 0.120 mm, #4: 0.121 mm.
(WMV)

Video S3 **Example jumps of** ***Acartia tonsa*** **played in slow motion X250.** Body lengths #1: 0.303 mm, #2: 0.280 mm, #3: 0.318 mm, #4: 0.138 mm.
(WMV)

Video S4 **Example swimming sequences of** ***Temora longicornis*** **played in slow motion X50.** Body lengths #1: 0.274 mm, #2: 0.215 mm, #3: 0.170 mm, #4: 0.168 mm, #5: 0.331 mm, #6: 0.350 mm.
(WMV)
